# On the Treatment of Enlarged Spleen

**Published:** 1852-06

**Authors:** W. Y. Gadbury

**Affiliations:** Benton, Mississippi


ART. II.—*On the Treatment of Enlarged Spleen.* By W. Y. GADBURY, M. D., of Benton, Mississippi.

The frequent occurrence of enlarged spleen renders it an object of special interest to the southern practitioner. Its intimate connection with the other viscera of the abdomen by means of the portal circulation; the elasticity of its tissues, the great relative size and horizontal course of its vessels, and the diminished ratio of mortality resulting from disease in it when compared with other organs of the animal economy, all indicate a wise provision of nature for the protection of the more important secretory organs from great influxes of blood in disease. The diagnosis and pathology of enlarged spleen are familiar to all, requiring no description here; but that its treatment is frequently unsuccessful is proved by the following quotation from standard authors. Dr. Dickson says, "the treatment of this disease is unsettled and by no means successful. When it becomes chronic our task is extremely difficult." Prof. Drake, whose experience in this disease entitles his opinion to more weight than that of any other author, says, in his great work on *Diseases of the Valley of North America*, that "in many instances it is impossible to reduce enlarged spleen while the patient continues in the locality where it originated."

These observations doubtless reflect the opinions of a majority of the profession. The failure to effect a cure, in many instances, is owing probably to oversight of the complications attending its progress. It is frequently associated with dyspepsia, with derangement of the hepatic and renal functions, and with general cachexia, anasarca,

&c. I lately had the care of a case which resisted all treatment until after the administration, empirically, of a vermifuge, when my patient was relieved after the discharge of fifty or more lumbricoides.

The depraved condition of the system generally contraindicates depletion as recommended by some authors. Purgatives are sometimes inadmissible, on account of diarrhea, and even when the bowels are torpid their continuance for a sufficient length of time to effect a cure by revulsion, may establish an irritation in the alimentary canal more intractable than the original disorder, particularly in southern latitudes. The frequent use of mercury is to be deprecated, as it aggravates the general cachexy The internal exhibition of the preparations of iodine is a practice highly extolled, but I have seen them fail oftener than succeed, and a persistence in their use will almost certainly bring on an unhealthy condition of the primæ viæ. They are sometimes serviceable in strumous habits where the bowels are healthy.

Quinine, that great panacea of the south, often succeeds in relieving an enlarged spleen before fibrinous depositions take place; after this it loses its controlling influence over this organ to a great extent. Its efficacy in suspending intermittent paroxysms ought not to be lost sight of as a preparatory step for a radical cure. The following combination will generally succeed in recent cases: ℞. Sulph. quinine, ʒj. Aquæ font. ℥viii., Acid. sulph, aromat. ʒij.: mix and add muriate of ammonia and cit. potass, áá. ʒij. Dose, a tablespoonful morning, noon, and night. The muriate of ammonia renders it so unpleasant to take, that patients can seldom be persuaded to continue its use long at a time; but, it may be remarked, this is no great objection to its use in hospital practice, or among our slave patients, where degustation is not much consulted.

Chalybeates are justly celebrated for their efficacy in this disease, and their union with alteratives and depuratives would seem to be indicated. Some four years since a case presented itself, which had resisted every method of treatment for two years, and in which I was induced to try the Liquor Oxysulphas Ferri, according to a recipe in the fifth number of Braithwait's Retrospect, (page 68) which is as follows:

℞. Ferri sulphat, ʒij. to ʒiij., Acid. nitric. ʒiij., Aquæ destillat. ℥iss.: rub the nitric acid with the sulphate of iron for fifteen minutes; then add the distilled water. The patient was ordered to take ten drops gradually increased to fifteen three times per day in a wine glassful of water. After the continuance of this remedy for two months, the chills ceased and have not as yet returned. The spleen under its use returned to its natural size. The successful treatment of this case induced me to try the following combination, which has proved universally successful when its use was continued for four or six weeks:

℞. Liquor Oxysulphat. Ferri ʒiv., Aquæ Cinnamomi, ℥viij., Sulphat. Quin., ʒj.: mix, and add cit. potass ʒij. to ʒiij. Dose, a tablespoonful morning, noon, and night. The dose is diminished as the cure progresses. During the administration of this remedy the patient is required to rub daily the splenic region with an ointment made by mixing iodine and basilicon ointment, over which is to be applied a flannel bandage, made as a lace jacket, and to be tightened every day; suspenders should be attached to prevent its rolling upon itself, and make it agreeable to wear. This gives support, and excites the absorbents to action. If the bowels are torpid, the patient may take Pil. Hydrarg, grs. x., Comp. ext. coloycinth, grs. v., for two or three nights, and afterwards regulate their action with soap, aloes, and rhubarb.

This *spleen* mixture is peculiarly adapted to dyspeptic patients, in no instance in my practice having disagreed with the bowels, but on the other hand regulating them when torpid without any perceptible purgative effect. It has the combined properties of a hepatic and renal emulgent, atiperiodic, alterative and tonic. Forty inveterate cases were thus successfully treated during the last two years, and I have yet to hear of an instance where a relapse has taken place. Under its use the chills cease to return, the digestive organs regain their natural powers, and the swarthy hue, so common in this disease, gives place to the ruddy complexion of health ; the nervous system is invigorated and the man is again himself.

April, 1852.

